# Health disinformation: a call to action for the hemostasis and thrombosis community

**DOI:** 10.1016/j.rpth.2025.103272

**Published:** 2025-11-19

**Authors:** David M. Smadja

**Affiliations:** 1 Université Paris Cité, INSERM PARCC, Team 7 "Endotheliopathy and Hemostasis Disorders", Paris, France; 2 AP-HP, European Georges Pompidou Hospital, Hematology Department, Paris, France

**Keywords:** artificial intelligence, communication, COVID-19, disinformation, education

## Abstract

The COVID-19 pandemic has exposed a parallel crisis: an infodemic of unprecedented scale and impact. This wave of misinformation and disinformation—particularly around thrombosis, vaccines, and hemostasis—has undermined public health measures, eroded institutional trust, and endangered scientific credibility. Within this hostile informational ecosystem, fringe theories proliferated across social media, falsely linking COVID-19 vaccines to widespread thrombosis via misinterpretations of D-dimer levels and anecdotal claims unsupported by clinical evidence. Political agendas, emotional manipulation, and algorithm-driven amplification created fertile ground for disinformation to thrive—especially among vulnerable populations like youth and vaccine-hesitant communities. The article dissects how belief bias, trust shortcuts, groupthink, and platform algorithms contributed to the viral spread of falsehoods about thrombosis and hemostasis. Case studies include the misuse of hydroxychloroquine, antivaccine conspiracy movements, and the misleading portrayal of rare vaccine-induced clotting events. The consequences were severe: declining vaccine uptake, harassment of scientists, and growing skepticism toward evidence-based medicine. This article calls on the hemostasis and thrombosis community to move beyond passive dissemination of knowledge. It proposes a 4-pillar strategy: embedding scientific voices in digital discourse, reforming media and health literacy education, enforcing stronger regulatory frameworks, and institutionalizing collective scientific engagement in mainstream and social media. As future health crises loom, communication must become as central as research itself. The article argues that the next battle for public health will be waged not only in hospitals and laboratories but also in the information spaces where truth competes with virality. It is time for science to go viral—for the right reasons.

## 1 Introduction

The COVID-19 pandemic not only overwhelmed health care systems but also unleashed a parallel and equally destructive crisis: an infodemic [1,2]. The infodemic—an overload of accurate and false information—revealed society’s vulnerability in processing truth, threatening public health, democracy, and trust in institutions [3]. With >90% of the occidental population owning smartphones [4], two-thirds of connected Westerners using social media daily, and 23% of 18- to 25-year-olds worldwide that getting their news from TikTok [5], health communication has shifted. In a Reuters study, health information ranks first among the interests of the French population, with 71% citing it as their primary area of concern [6]. This infodemic, fueled by bias, politics, and algorithms, reveals that pandemic response needs more than science—it needs clear communication and public trust. A new kind of power has emerged—algorithmic power—but without the necessary counterbalances.

This article explores the anatomy of this infodemic, using the COVID-19 pandemic and hemostasis concerns as a case study. It analyzes the social, psychological, and technological drivers of health disinformation [7], evaluates its impact on public behavior and trust, and proposes a forward-looking strategy to combat current and future infodemics.

## 2 The Rise of the Infodemic During Covid-19

**Figure 1:**
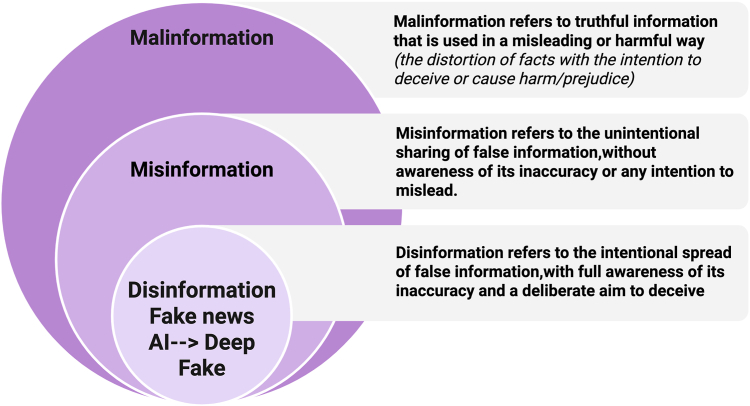
Classification of information disorders: malinformation, misinformation, and disinformation. This figure illustrates the conceptual distinctions between the 3 main types of information disorders. Malinformation, while based on truthful content, is deliberately used out of context or distorted to cause harm or prejudice. These categories help clarify the intent and veracity dimensions in information integrity assessments. Misinformation refers to the unintentional dissemination of false content, often shared without awareness of its inaccuracy. Disinformation involves the intentional spread of false information with the aim to deceive or manipulate (eg, hate speech and harmful rumors). Created in BioRender. Smadja D. (2025) https://BioRender.com/19yfw50.

Malinformation (Figure 1), misinformation, and disinformation are forms of information disorder [8]. Disinformation, in particular, has evolved in scale and speed due to artificial intelligence (AI) deepfakes, fake news, and algorithmic amplification—posing threats to trust, democracy, and social cohesion. Democracy is like a muscle: it must be exercised and strengthened by everyone. The general population need to train it collectively or risk letting it weaken in the face of automated influence. The COVID-19 crisis demanded that researchers and health professionals become high-performance athletes in public communication—constantly adapting, informing, and engaging a population under pressure.

The COVID-19 pandemic was the first global health crisis in the digital age, triggering what World Health Organization called an infodemic [9]—an overwhelming surge of both true and false information. While health care systems tackled SARS-CoV-2, disinformation on platforms like Twitter, Facebook, and TikTok undermined public health efforts. Evidence-based medicine, defined by Sackett et al. [10] as the use of best current evidence in patient care, clashed with media narratives dominated by emotion, anecdotes, and speed. A key case was the hydroxychloroquine (HCQ) controversy. Although clinical trials disproved its efficacy [11,12], HCQ was promoted by leaders and conspiracy theorists [13]. A French daily newspaper on April 5, 2020, even polled the public on HCQ effectiveness—reflecting the collapse between science, generalist media, and public belief. Indeed, public endorsements had measurable impact. A US study showed that after Elon Musk and President Trump endorsed HCQ (March 2020), Google searches for buying HCQ spiked by 1389%, and chloroquine by 442% [14]. Cable news also shaped health behavior. One study linked higher Fox News viewership to lower COVID-19 vaccination rates, especially in those under 65 years, unrelated to general vaccine attitudes or access [15]. Yale research further showed Republican voters had a 15% higher excess death rate in states like Ohio and Florida [16]. This phenomenon of antiscience sentiment within the Republican Party has intensified significantly since 2024’s presidential campaign rhetoric [17]. Disinformation also skewed scientific understanding. Didier Raoult publicly cited the number of views on his YouTube videos as a measure of scientific credibility, positioning this as an alternative to conventional academic rankings such as the Shanghai ranking. Based on the popularity of his videos, he went so far as to claim that his research institute of infectious disease surpassed the whole Harvard University in academic influence. In the same vein, during a media interview, he stated, “I must say that most of the time, the information on YouTube is of better quality than in traditional media,” referring to the content he regularly published on his channel [18].

In hemostasis, the protein D-dimer became a disinformation target. Although useful in assessing clotting risk in COVID-19 [[19], [20], [21], [22]], some falsely claimed postvaccine D-dimer increases proved widespread thrombosis. Individuals like Dr Hoffe in Canada circulated unverified claims, and outlets like The Epoch Times [23,24] distributed them during antivaxx protests in Paris. In a nationwide French study of million’s hemostasis tests (2013-2021), our team found that while most testing declined in 2020 due to lockdowns, D-dimer testing sharply increased [25]. In March 2021, our early pharmacovigilance data showed no increase in thrombotic events after vaccination start—just 0.21 cases per million vaccinated person-days, lower than expected [26]. Major vaccines like Pfizer and Moderna showed no thrombotic risk compared with COVID-19 itself [27] and did not cause coagulation disorders [28]. Rare cases of vaccine-induced thrombotic thrombocytopenia were linked to adenovirus-based vaccines and described by *New England Journal of Medicine* papers and several case reports [[29], [30], [31]], suggesting an immunological mechanism due to adenovirus used to make the vaccine rather than a generalized clotting risk [29] and give rise to the birth of a new thrombotic autoimmune disorders [30,32]. Yet, antivaccine influencers weaponized these rare events, claiming, falsely, that blood donations were unsafe postvaccine or that spike proteins caused permanent damage. Peer-reviewed rebuttals were sometimes ignored, while flawed studies in predatory journals remained online, fueling disinformation [33]. Moreover, antivaccine influencers and pandemic disinformers often aligned themselves with various forms of conspiracy thinking, including antisemitism and antiestablishment rhetoric. In France, former Health Minister Agnès Buzyn was portrayed in antisemitic cartoons. I, myself, was depicted wearing a yellow star, a grotesque appropriation of the symbol used to persecute Jews during World War II. Disinformation made vaccination an identity marker. In some countries, political ideology—not science—predicted vaccine behavior. Trust in institutions like the Centers for Disease Control and Prevention dropped from 69% to 44% (2020-2022), while inconsistent messaging worsened public confusion. Psychological biases helped disinformation spread. Belief bias led people to accept claims from trusted or charismatic figures over scientific evidence. Heuristics like imitation and trust bias explain why fringe figures or fact gained influence. Platforms like YouTube, Facebook, and Twitter/X amplified emotionally charged content. For instance, early COVID-19 reports in *New England Journal of Medicine* suggested links between antiphospholipid antibodies and coagulopathy—but were based on only 3 cases. Later studies [34,35] showed no association with thrombosis but received less attention. Research by Vosoughi et al. [36] found that false news spreads faster and deeper than truth—70% more likely to be retweeted, with the top 1% of false stories reaching over 100,000 people. A meta-analysis of 14 randomized controlled trials (18,000+ participants) confirmed exposure to health disinformation increases belief in false claims. Notably, longer messages (>100 words) had greater impact [37]. Generative AI has worsened the problem. AI-generated messages are 64% more persuasive than human-written ones when tailored to individuals [38]. These tools mass-produce false claims, deepfakes, and even pseudoacademic papers. Young people are particularly vulnerable, spending an average of 30.6 hours per week on their smartphones [39]. With universities withdrawing from social platforms by 2025 [40], a vacuum was filled by influencers and conspiracy theorists. During COVID-19, astroturf campaigns used bots to fake grassroots vaccine resistance, leveraging social proof bias. Engagement-driven algorithms amplified emotional disinformation over accuracy. This systemic issue allowed fringe papers and fake journals to outperform peer-reviewed science. Automated networks of bots further flooded platforms with antivaccine narratives. The consequences are measurable. False claims about D-dimer, spike protein, and vaccine toxicity led to consequences such as lower vaccine uptake, delayed boosters, and rising ICU admissions. In France, AstraZeneca’s vaccine temporary suspension in March 2021—although scientifically justified—sparked public panic. Medical experts faced harassment. Scientific truth struggled to compete with viral lies. Disinformation’s danger lies not just in its content, but in its delivery—emotionally resonant, repetitive, and identity affirming. Ultimately, defeating medical disinformation demands more than facts. It requires strategic communication, emotional storytelling, and a deep understanding of human psychology and digital dynamics.

## 3 Recommendations: How to Fight Medical Disinformation

Having seen the devastating effects of medical disinformation during the COVID-19 pandemic—from vaccine refusal to institutional distrust—it is clear that countering falsehoods cannot be an afterthought in public health. What researchers and health professionals now face is not just a battle for facts but also a strategic, long-term campaign to defend truth, trust, and science. The COVID-19 pandemic revealed many vulnerabilities in our global health systems, but none as surprising—or as dangerous—as our fragility in the face of disinformation [3]. Hemostasis and thrombosis have been in the center of pandemics and so in the center of disinformation during this period. From conspiracy theories about vaccines and D-dimer levels to orchestrated online movements designed to sow doubt, the dual pandemics—of disease and disinformation—have reshaped how the public engages with science, medicine, and authority. Disinformation is not new. The use of misleading narratives to manipulate opinion dates back to ancient times—from libellees in the Enlightenment to wartime propaganda. But today, these tactics have been amplified, automated, and weaponized by technology. Through deepfakes, algorithmic virality, astroturfing, and AI-generated pseudoscience, disinformation has become a global, scalable threat. Throughout this article, I have explored the multifaceted nature of the infodemic: from psychological vulnerabilities and social media architecture to political manipulation and institutional withdrawal [40]. To effectively prepare for—and ultimately prevent—the next health worldwide emergency, the scientific and medical community must recognize communication not as an auxiliary function, but as a core strategic priority: The Next Pandemic Is a Question of “When,” Not “If.” The infodemic experienced during COVID-19 will not be the last. Whether it is another virus, a climate emergency, or a large-scale technological disruption, the next global crisis will again test our ability to communicate quickly, clearly, and credibly. Communication must be seen as central to the mission of science. Rather than remaining on the sidelines, science must fully enter the public arena, telling its stories with the same clarity, emotion, and resonance as those who spread disinformation. Health education must be accessible, engaging, and viral—like entertainment or conspiracy theories. Institutions must rejoin platforms like Twitter/X, TikTok, Instagram, YouTube, and AI tools to meet people where they are.

**Figure 2:**
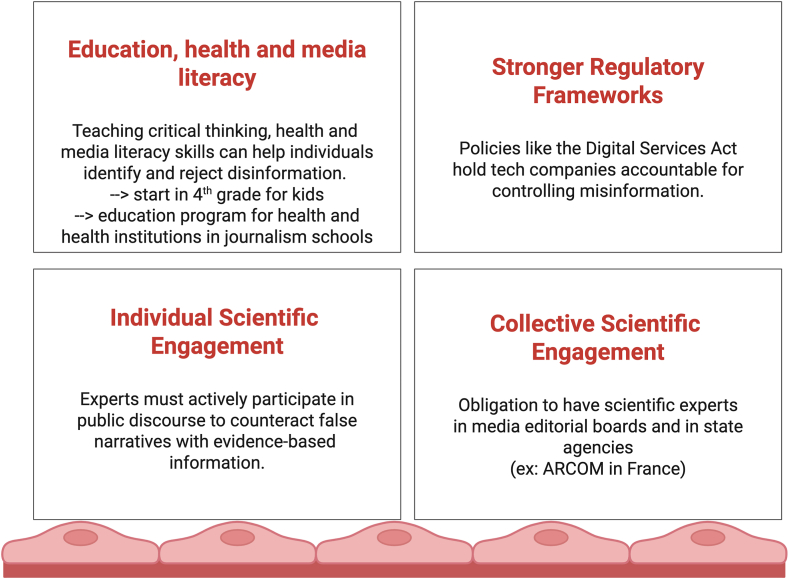
Combating disinformation: a call to action across knowledge, technology, and policy. This figure presents a multidimensional strategy to confront and mitigate the spread of disinformation. It emphasizes the need for critical media literacy among the public, robust fact-checking mechanisms, and the ethical regulation of digital platforms. Key pillars include education and empowerment through digital, health, and media literacy; policy and governance via regulatory frameworks that ensure accountability while preserving democratic freedoms; and collaborative ecosystems that foster cooperation among governments, technology providers, academia, and civil society. Together, these coordinated efforts aim not only to detect and dismantle disinformation but also to build societal resilience against future infodemics. Created in BioRender. Smadja D. (2025) https://BioRender.com/lucnn6h.

Figure 2 proposes 4 strategic pillars to counter medical disinformation. This figure outlines 4 coordinated levers of action essential to rebuilding trust in public health and countering the spread of medical disinformation on digital platforms.

### 4.1 Education, health, and media literacy

Empowering individuals through early and structured education is a long-term solution to disinformation. Teaching critical thinking and health/media literacy from as early as 9 to 10 years old helps future generations identify and reject false health narratives. More than health, a focus needs to be done to health institutions. Moreover, it is essential to integrate more media and scientific/health literacy programs into journalism training curricula.

### 4.2 Stronger regulatory frameworks

Policies like the Digital Services Act in Europe exemplify how governments can hold platforms accountable. These frameworks must ensure technology companies are actively responsible for monitoring, moderating, and reducing the spread of health-related disinformation.

### 4.3 Individual scientific engagement

Scientists and clinicians must take part in public discourse—not only within academic journals but also across social media, mainstream platforms, and media. Their presence helps to challenge viral falsehoods with evidence-based information and restores visibility to verified science in digital spaces.

### 4.4 Collective scientific engagement

Governments, institutions, and public agencies must embed scientific expertise into their organization, their media strategies and editorial oversight. This includes a structural obligation to include scientific advisors on editorial boards of all kinds of media and within public regulatory bodies (eg, ARCOM in France), ensuring that media content adheres to standards of scientific accuracy and public health relevance.

All in all, a resilient society equips not just its institutions, but the minds of its people. COVID-19 revealed how easily truth is outrun by myth. Its legacy should be one of adaptation, not confusion—because in an age of information overload, discernment is vital. Emerging challenges in thrombosis and hemostasis—such as glucagon-like peptide 1– or immunotherapies-related vascular risks, new hemophilia therapies, and cancer-associated thrombosis in a world where cancer is largely outpatient—must be met with exemplary communication. These issues affect millions and unfold in a media landscape far removed from the past. The International Society on Thrombosis and Haemostasis must take this challenge seriously. We are all contributors to the science that drives this organization—but that is no longer enough. All researchers and health professionals must also become its ambassadors. Not just to our students and peers, but to the broader public, to mainstream media, to social platforms, and to public decision-makers. This demands far more than a few community managers. It will require bold, sustained investment in large-scale public communication strategies. Our community need professional science influencers—before, during, and after our major International Society on Thrombosis and Haemostasis congresses. Let us make it happen—starting in Paris, July 2026.
